# Mdig de-represses H19 large intergenic non-coding RNA (lincRNA) by down-regulating H3K9me3 and heterochromatin

**DOI:** 10.18632/oncotarget.1155

**Published:** 2013-08-10

**Authors:** Bailing Chen, Miaomiao Yu, Qingshan Chang, Yongju Lu, Chitra Thakur, Danjun Ma, Zhengping Yi, Fei Chen

**Affiliations:** ^1^ Department of Pharmaceutical Sciences, Eugene Applebaum College of Pharmacy, Wayne State University, 259 Mack Avenue, Detroit, MI, USA

**Keywords:** mdig, H19, demethylation, prognosis

## Abstract

Mineral dust-induced gene (mdig) had been linked to the development of human lung cancers associated with environmental exposure to mineral dust, tobacco smoke or other carcinogens. In the present studies, we demonstrated that the overexpression of mdig in A549 adenocarcinomic human alveolar type II epithelial cells decreases the heterochromatin conformation of the cells and de-represses the transcription of genes in the tandemly repeated DNA regions. Although mdig can only cause a marginal decrease of the total histone H3 lysine 9 trimethylation (H3K9me3), a significant reduction of H3K9me3 in the promoter region of H19, the paternally imprinted but maternally expressed gene transcribing a large intergenic non-coding RNA (lincRNA), was observed in the cells with mdig overexpression. Silencing mdig by either shRNA or siRNA not only increased the level of H3K9me3 in the promoter region of H19 but also attenuated the transcription of H19 long non-coding RNA. Demethylation assays using immunoprecipitated mdig and histone H3 peptide substrate suggested that mdig is able to remove the methyl groups from H3K9me3. Clinically, we found that higher levels of mdig and H19 expression correlate with poorer survival of the lung cancer patients. Taken together, our results imply that mdig is involved in the regulation of H3K9me3 to influence the heterochromatin structure of the genome and the expression of genes important for cell growth or transformation.

## INTRODUCTION

Mineral dust-induced gene (mdig) was first identified in people with chronic lung diseases resulting from occupational exposure to mineral dust in the mining industry [1, 2]. This gene was independently identified in human glioblastoma cell line T98G cells that overexpress the c-Myc oncogene and named as myc-induced nuclear antigen 53 (mina53) [3]. In addition, mdig was found to be a constitutive nucleolar component in a number of cell types from *Xenopus laevis* to humans and was also named nucleolar protein 52 (NO52) [4]. An elevated expression of mdig has been observed in some human cancers, including lung cancer [2], colon cancer [5], esophageal squamous cell carcinoma [6], gingival squamous cell carcinoma [7], lymphoma [8], renal cell carcinoma [9], neuroblastoma[10], gastric cancer[11], hepatocellular carcinoma[12], and cholangiocarcinoma [13]. The mdig protein contains a conserved JmjC domain that was found in the family of Fe(II) and 2-oxoglutarate (2OG)-dependent dioxygenase enzymes that serve as histone demethylases [14]. Our previous study implicated that mdig is involved in reducing the trimethylation of histone H3 lysine9 (H3K9me3) in the human bronchial epithelial cell line, BEAS-2B [2]. This notion was supported by the fact that the increased expression of mdig is inversely correlated with the level of H3K9me3 in the majority of human lung cancer samples examined. In addition, evidence suggests that mdig contributes to cell growth regulation, possibly by promoting cell cycle entrance from G1 phase to S phase and/or the synthesis of rRNA [1-3]. Furthermore, mdig had been shown to be capable of affecting gene expression outside the nucleolus, for example, by repressing IL-4 expression in T helper cells [15]. Most recently, mdig has been implicated as a 2-oxoglutarate oxygenase that is responsible for the histidyl hydroxylation of Rpl27a, a subunit of the 60S ribosome that is important for protein translation [16].

H19 is a paternally imprinted but maternally expressed oncofetal gene transcribing a large intergenic non-coding RNA (lincRNA) and is located on chromosome 11p15.5 in humans. During the earlier period of embryogenesis, both paternal and maternal H19 alleles are expressed. In most adult tissues, however, the paternal H19 allele is silenced due to the formation of a high order heterochromatin structure in the H19 gene locus [17, 18] as a result of both DNA methylation and H3K9me3 [19, 20]. The role of H19 in cancer development has been a matter of debate since its discovery in 1990 [21-23]. The possible tumor suppressor-like function of H19 was derived from the observed tumor growth inhibition resulting from the H19 cDNA transfection of G401-transformed kidney cells and the fact that H19 serves as a precursor of microRNA-675 (miR-675) that targets insulin-like growth factor 1 receptor (IGF1R) [24, 25]. However, several lines of evidence suggest that H19 is more likely an oncogene, rather than a tumor suppressor. First, a number of human cancers, including hepatocellular carcinoma, lung cancer, esophageal cancer, colorectal cancer, bladder cancer, prostate carcinoma, Ewings' sarcoma, and germ cell carcinoma, exhibited increased expression of H19 [22]; secondly, the ectopic expression of H19 enhances the tumorigenicity of breast cancer cells [26]; thirdly, silencing H19 by siRNA attenuated the clonogenicity and anchorage-independent growth of the lung cancer cells and breast cancer cells [27]; lastly, the expression of H19 is up-regulated by both c-Myc and E2F1 oncogenes [23, 27].

We previously reported an increased expression of H19 in hepatocellular carcinoma with higher JNK1 activation [28, 29]. Because mdig has been implicated in the regulation of rRNA genes, many of which are repressed due to the heterochromatin conformation of the gene loci [30, 31], we aimed to explore whether mdig is also capable of regulating the expression of genes in tandemly repeated DNA regions, such as H19, a lincRNA that is not expressed in adult tissues as a result of the highly condensed heterochromatin structure in the promoter and the differentially methylated region (DMR)/ imprint control region (ICR) of the H19 gene. Our data showed that mdig enhances H19 expression by reducing the level of H3K9me3 in the promoter and ICR region of the H19 gene. The *in vitro* demethylation assay using immunoprecipitated mdig protein and histone H3 peptide containing H3K9me3 indicated that the mdig is able to demethylate H3K9me3. These findings may thus provide a new mechanistic insight into the oncogenic role of the mdig gene in the development of human cancers.

## RESULTS

### Mdig reduces nuclear heterochromatin staining by DAPI

**Figure 1:**
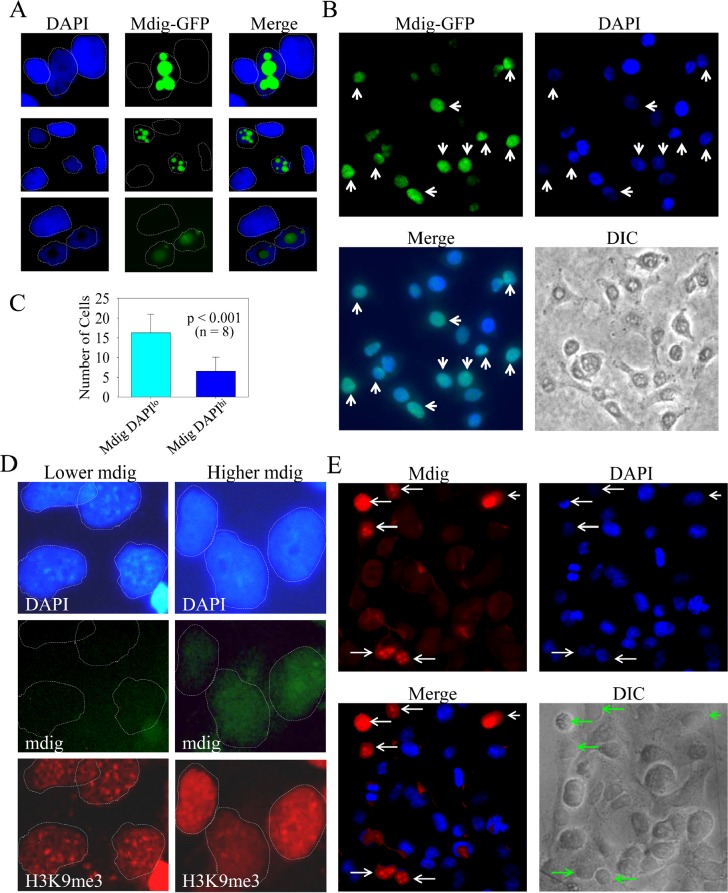
Mdig expression reduces heterochromatin staining A. Cells expressing mdig-GFP showed reduced DAPI staining; B. A randomly selected field of microscopic image with lower magnification shows the inverse correlation of the GFP signal and the strength of the DAPI signal (arrows); C. Quantification of the cells expressing mdig-GFP with weaker DAPI staining and the cells expressing mdig-GFP with normal or strong DAPI staining; D. Mdig expression diminishes the heterochromatin foci as evidenced by the reduced number of bright DAPI-stained foci and H3K9me3 foci. Note the overlapping pattern of the bright DAPI staining foci and the H3K9me3 foci in the cells without mdig-GFP signal; E. Cells expressing a higher level of endogenous mdig showed reduced DAPI staining (arrows).

High levels of mdig expression have been found in a number of human cancers. In human lung cancers, our previous data showed an inverse correlation between the levels of mdig protein and H3K9me3 [2]. In the present studies, we initially aimed to determine the contribution of mdig to the growth regulation of the A549 adenocarcinomic human alveolar basal epithelial cells by establishing stably transfected cell lines overexpressing mdig-GFP protein. In a routine examination for the expression of mdig-GFP by fluorescence microscopy, we frequently noted that cells expressing mdig-GFP always showed a dimmed nuclear staining signal by 4',6-diamidino-2-phenylindole (DAPI), a fluorescent dye that binds strongly to repetitive A-T rich DNA (Fig. 1A). In a randomly selected microscopic field, the number of cells that showed mdig-GFP expression but dimmed staining of DAPI was much higher than the cells showing mdig-GFP expression and normal or higher DAPI staining (Figs. 1B and 1C). Under high magnification, heterochromatin foci that contain highly condensed and repetitive tandem DNA can be visualized by DAPI staining among the cells without any notable expression of mdig-GFP. These heterochromatin foci, however, were absent in the cells expressing mdig-GFP (Fig. 1D). Because it is known that H3K9me3 is enriched in the heterochromatin region [33], we also determined the effect of mdig on H3K9me3 in A549 cells by fluorescent microscopy. Indeed, a similar pattern of H3K9me3 foci that are roughly overlapping with the heterochromatin foci as stained by DAPI was seen in the cells without mdig-GFP. In contrast, these H3K9me3 foci were faint in the mdig-GFP expressing cells (Fig. 1D, bottom panels).

To exclude the possibility that the above observation was a result of the overexpression of the exogenous protein that might have caused an artificial reduction of the DAPI staining signal, we next evaluated the effect of endogenous mdig on DAPI staining. The level of endogenous mdig was measured by staining the cells with an anti-mdig antibody and a secondary antibody conjugated with Alexa Fluor 680 that emits a red fluorescent signal under fluorescent microscopy. A similar inverse correlation of the levels of endogenous mdig and DAPI staining was observed (Fig. 1E, compare the cells indicated by white arrows to the cells without arrow markers). These data clearly indicate that the expression of mdig reduces the overall level of heterochromatin conformation.

### Marginal effect of mdig on the total H3K9me3 in A549 cells

**Figure 2:**
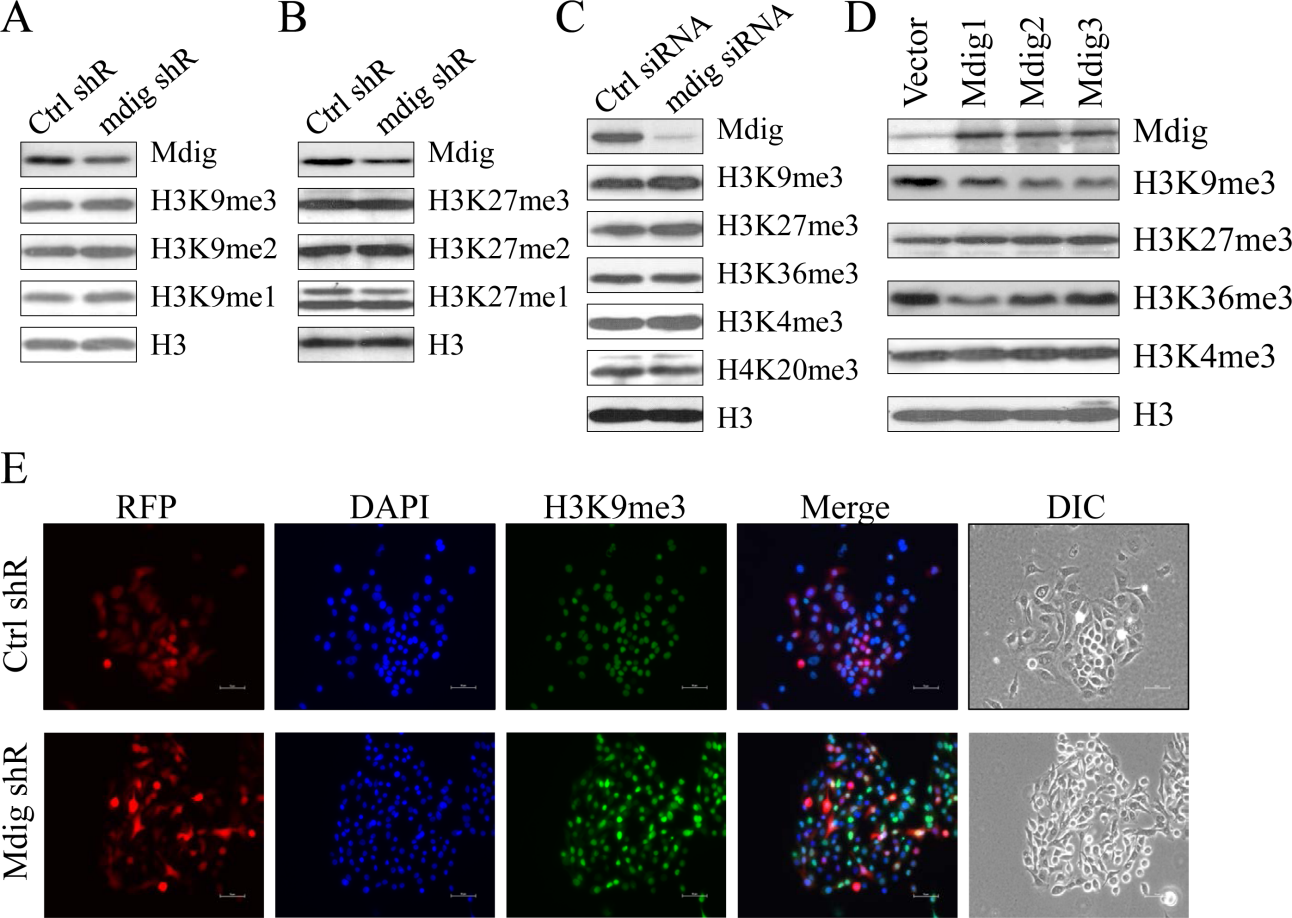
Marginal effect of mdig on total H3K9me3 A & B. Western blotting detection for the mdig protein and the histone H3 methylation status in the cells stably transfected with control shRNA or mdig shRNA. C. Western blotting detection of the mdig protein and the histone H3 methylation status in the cells transfected with a control siRNA or mdig siRNA. D. Western blotting detection of the mdig protein and the histone H3 methylation status in the cells stably expressing a control vector or mdig. E. Immunofluorescent staining of H3K9me3 in the cells stably expressing an RFP-tagged control shRNA or an RFP-tagged mdig shRNA. Nuclei were staining by DAPI.

The mdig protein contains a conversed JmjC domain, the signature motif of the histone demethylase family of proteins. Although previous studies showed a reduced level of H3K9me3 in the BEAS-2B cells transfected with mdig, direct evidence regarding whether mdig is a histone demethylase is still lacking. In the stably transfected A549 cells that express mdig shRNA that silences mdig, we noted about a 50 to 60% reduction of mdig protein. However, only a very marginal increase of H3K9me3 and H3K9me1 was observed in these cells (Fig. 2A). No notable effect of mdig on the methylation states of H3K27 was observed (Fig. 2B). To exclude the possibility that the shRNA had a poor silencing effect on mdig in the stably transfected cells, we also used siRNA to silence mdig in A549 cells. Although the siRNA showed much improved efficacy on silencing mdig (Fig. 2C), again, only a marginal increase of H3K9me3 was detected.

To further examine the potential role of mdig on H3K9me3, we evaluated the methylation status of histone H3 protein in several clones of the cells stably expressing mdig. All three mdig stable expression clones showed some degree of reduction in the level of H3K9me3 as compared to the clone stably expressing a control vector (Fig. 2D). No conclusive effect of mdig overexpression on H3K27me3, H3K36me3 and H3K4me3 was observed. Consistent with the result of immunoblotting on the effect of mdig shRNA on H3K9me3 (Fig. 2A), immunofluorescent staining also indicated a marginal increase of H3K9me3 in the cells stably expressing mdig shRNA (Fig. 2E).

### Mdig de-represses the transcription of genes in heterochromatin regions

**Figure 3:**
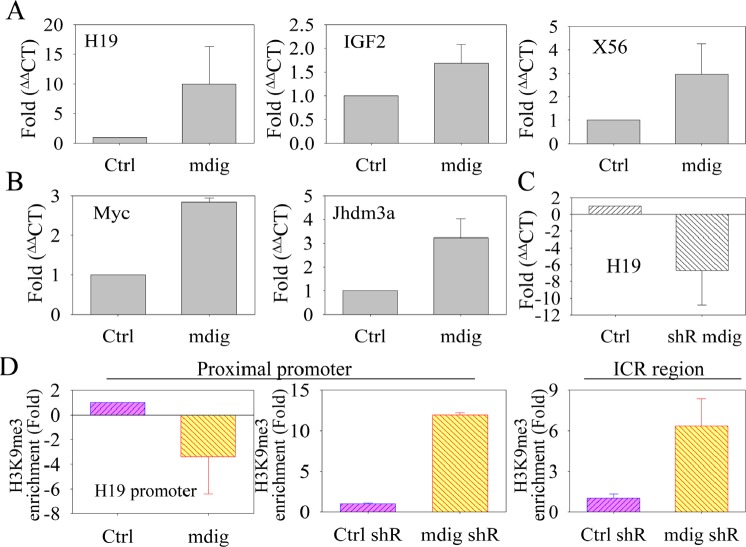
Mdig enhances the expression of genes in the heterochromatin region A. Quantitative real-time PCR showing that mdig overexpression increases the expression of H19, IGF2 and macrosatellite X56, which are normally repressed by the heterochromatin conformation. B. Quantitative real-time PCR showing that mdig upregulates the expression of c-Myc and Jhdm3a. C. Quantitative real-time PCR showing that silencing mdig by shRNA suppressed the expression of H19. D. Chromatin immunoprecipitation (ChIP) assays showing that mdig overexpression reduces the enrichment of H3K9me3 in the promoter region of the H19 gene (left panel). Silencing mdig by shRNA increased the enrichment of H3K9me3 in the promoter (middle panel) and the ICR region (right panel) of the H19 gene.

Our immunoblotting data showed that mdig only exhibited a very marginal effect on the total H3K9me3 level in the cells in which mdig is overexpressed or silenced by siRNA or shRNA (Fig. 2). One possibility of such a weak effect may be a result of the limited activity of mdig on the global level of H3K9me3 which is distributed in a wide range of chromatin types, including constitutive heterochromatin, facultative heterochromatin, repressive and non-repressive euchromatin, etc. To determine the potential influence of mdig on a limited scale of epigenetic regulation, we focused on the effect of mdig on the transcription of genes that are known to be silenced in the heterochromatin regions, such as satellite and imprinting loci. H19 and IGF2 are paternally and maternally imprinted genes, respectively, due to the highly repetitive DNA elements between these two gene loci and the developmental formation of a highly condensed heterochromatin structure in their gene loci. Quantitative RT-PCR experiments showed that the transcript levels of H19 and IGF2 were elevated 9.98-fold and 1.7-fold, respectively, in the cells that stably overexpressed mdig (Fig. 3A). In addition to H19 and IGF2, we also investigated the level of a long non-coding transcript from the macrosatellite X56 that is packaged in facultative heterochromatin characterized by H3K9me3 and CpG DNA hypermethylation at the active X chromosome [34]. The overexpression of mdig induces a 2.96-fold increase of macrosatellite X56 transcript levels (Fig. 3A, right panel). Furthermore, 2.8-fold and 3.4-fold increases of c-Myc and Jhdm3a expression were observed in the mdig-overexpressing cells relative to the cells expressing a control vector (Fig. 3B). The increased expression of c-Myc may be important in mediating the growth-promoting feature of the mdig protein, whereas elevated Jhdm3a, a known H3K9me3 demethylase, may contribute to the demethylation of H3K9me3 and possibly H3K36me3 in the mdig-expressing cells [35].

Prompted by the highest induction of H19 among the transcripts examined in mdig-overexpressing cells, we next focused our attention on the level of H19 mRNA in the cells in which mdig was silenced by shRNA to validate the role of mdig on the de-repression of genes in the heterochromatin region. We assume that if mdig de-represses H19 as we observed in the mdig-overexpressing cells, silencing mdig may reduce the transcription of H19. As expected, silencing mdig resulted in a 6.2-fold reduction of the H19 transcript relative to the cells expressing a control shRNA (Fig. 3C).

The H19 gene is located in the short arm and near the end of chromosome 11 in humans. The expression of H19 is regulated by the methylation status of the DNA and histone protein in the promoter and the ICR of the H19 gene [17, 18]. ICR methylation and the formation of H3K9me3 on the nucleosomes in this region will prevent the binding of CCCTC-binding factor (CTCF) to the ICR and the intrachromasomal looping of the enhancer to the promoter region of the H19 gene, leading to the repression of H19 transcription [36]. To test the hypothesis that mdig de-represses H19 transcription by affecting the level of H3K9me3, we determined the abundance of H3K9me3 in the promoter and ICR of the H19 gene locus by chromatin immunoprecipitation (ChIP) experiments in mdig-overexpressing cells or cells in which mdig is silenced by shRNA. Indeed, the overexpression of mdig diminished the enrichment of H3K9me3 in the H19 gene promoter (Fig. 3D). In contrast, silencing mdig with shRNA increased the enrichment of H3K9me3 in the promoter and ICR by 12-fold and 6-fold, respectively, compared to the control shRNA (Fig. 3D, middle and right panels). These data clearly indicate that mdig is capable of de-repressing genes in the heterochromatin region by reducing the level of H3K9me3, although mdig has a marginal effect on the global level of H3K9me3, as indicated by Western blotting using total cellular proteins (Fig. 2).

### Mdig demethylates H3K9me3 on the histone H3 peptide

**Figure 4:**
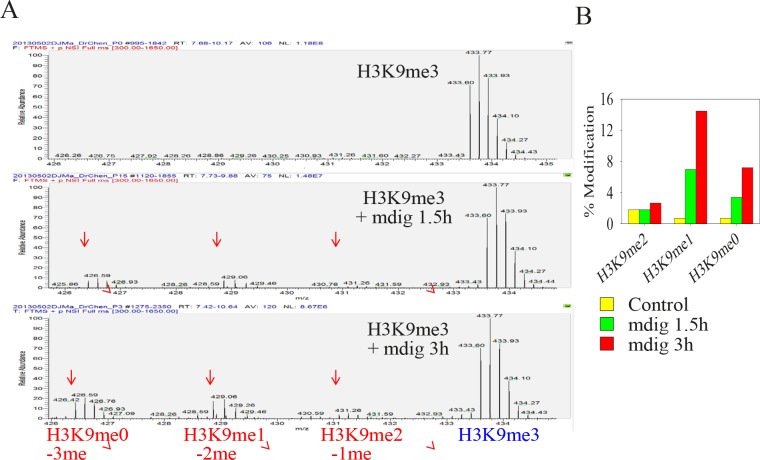
Immunoprecipitated mdig from mdig-GFP expressing cells can demethylate H3K9me3 A. Mass spectrum diagrams showing the mdig-mediated demethylation of H3K9me3. Top panel: incubation of H3K9me3-histone H3 peptide with control IgG; middle panel: incubation of H3K9me3-histone H3 peptide with immunoprecipitated mdig protein for 1.5 h; bottom panel: H3K9me3-histone H3 peptide with immunoprecipitated mdig protein for 3.0 h.B. Quantification of the demethylation products.

As a key regulator and epigenetic marker of heterochromatin, H3K9me3 has been extensively studied for its role in the conformation, maintenance and propagation of heterochromatin. The removal of H3K9me3 by histone demethylases decondenses the heterochromatin structure and de-represses the expression of genes in the heterochromatin region featured with tandemly repeated DNA. Uncertainties remain regarding whether mdig possesses histone demethylase activity based on some cellular and biochemical studies [2,16]. To gain direct evidence showing that mdig may contribute to the demethylation of H3K9me3, we incubated immunoprecipitated mdig from the lysates of mdig-overexpressing cells with a histone H3 peptide containing H3K9me3 for 1.5 and 3 h, respectively. Demethylation activity was then determined by tandem mass spectrometry. As shown in Fig. 4, the incubation of the lysine 9 tri-methylated histone H3 peptide for 1.5 h resulted in the appearance of H3K9me2, H3K9me1, and H3K9me0 peptides (Fig. 4A, middle panel, and Fig. 4B). The concentrations of these peptides with different methylation states were increased further when the time of incubation was increased from 1.5 h to 3 h (Fig. 4A, bottom panel, and Fig. 4B). The quantification of the demethylation activity of the immunoprecipitated mdig protein toward the histone H3 peptide substrate suggested that mdig is able to remove one, two and three methyl groups, with the strongest capability to remove two methyl groups from H3K9me3 (Fig. 4B). To exclude the possibility that such a demethylation was a result of contaminated cellular proteins due to non-specific binding in the immunoprecipitation step, we also performed this demethylation reaction using immunoprecipitated c-Jun, a known nuclear protein, or GAPDH, a cytosolic protein. No demethylation activity was detected when the immunoprecipitated c-Jun or GAPDH was included (data not shown).

### Increased expression of mdig and H19 is associated with poorer survival of lung cancer patients

**Figure 5:**
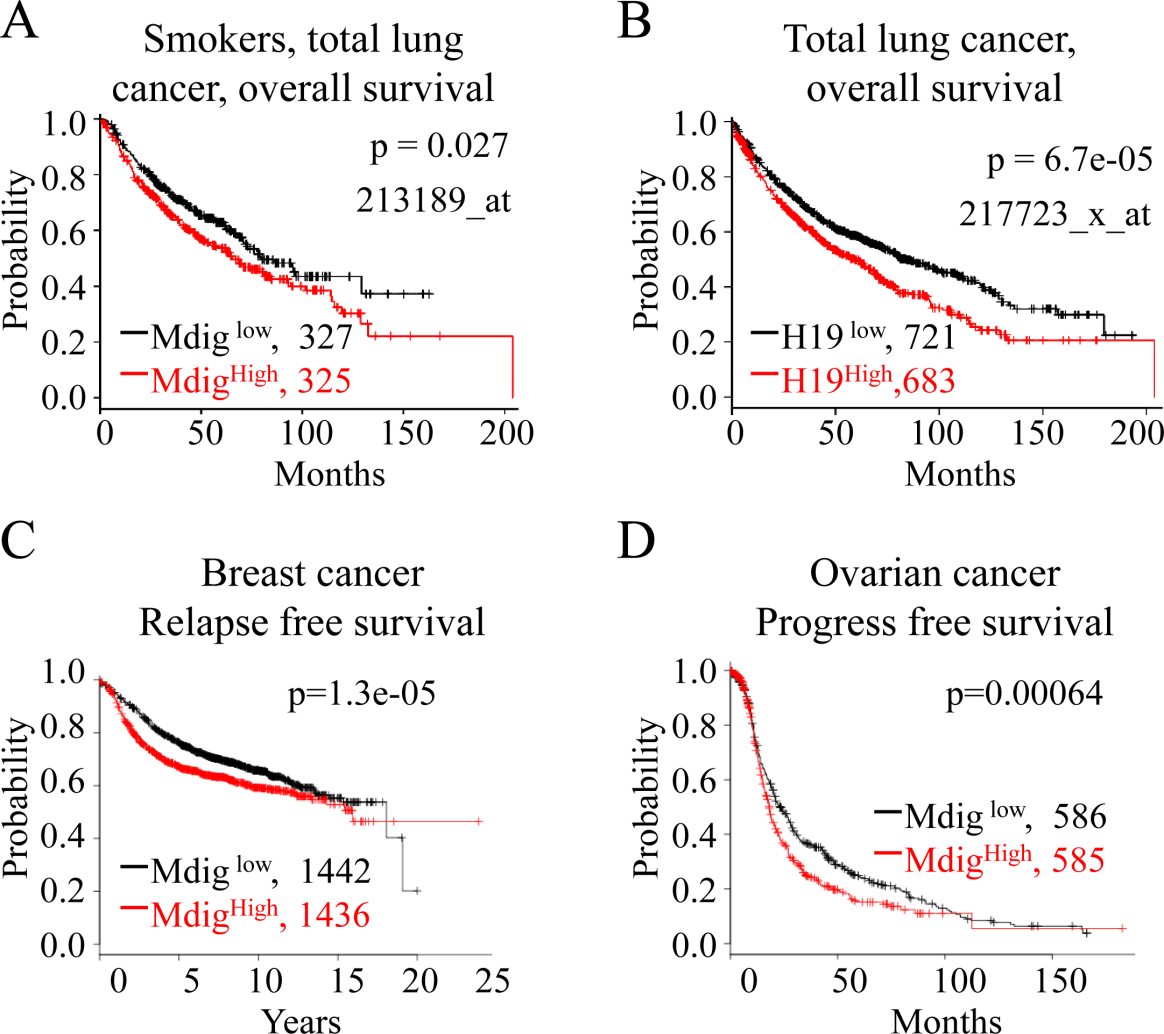
Higher expression of mdig and H19 is associated with poorer survival of cancer patients A. Kaplan-Meier plot showing poorer survival of the lung cancer patients who were smokers or former smokers and had higher expression of mdig mRNA. B. Higher level of H19 expression correlates with poorer survival of lung cancer patients. C & D. Higher level of mdig is associated with poorer relapse-free survival of the breast cancer patients and poorer progress-free survival of the ovarian cancer patients.

To investigate whether our findings mentioned above could be relevant to the clinical outcomes of human lung and other cancers, we examined the prognostic value of mdig and H19 in a clinical microarray database of lung cancer [37]. The database collected gene expression data that were obtained by using three different versions of Affymetrix HG-U133 microarrays and the survival information of 1,715 lung cancer patients. Two different mdig probe sets, probes 213188_s_at and 213189_at, are presented in this database. After careful analyses of the sequence information of these two probes, we found that the probe set 213188_s_at, in fact, detects the far end of the 3'-UTR of mdig mRNA and the antisense of 3'-UTR of the β-γ-crystallin domain containing 3 (CRYBG) mRNA. Thus, this probe set was not included in our assay. The probe set 213189_at, nonetheless, detects the open-reading frame (ORF) of mdig mRNA, which may much more accurately detect the true expression level of the mdig mRNA. Accordingly, we used this probe set for the correlation assay of mdig expression with the survival of lung cancer patients. Although the mdig level has no significant correlation with the survival of total lung cancer patients, we noted a strong association between a higher level of mdig with poorer overall survival of the lung cancer patients who were smokers or former smokers (Fig. 5A). Because we noted that mdig enhanced H19 expression by demethylating H3K9me3 in the promoter and ICR region of the H19 gene (Figs. 3 and 4), we next aimed to determine whether higher levels of H19 also predict a poorer prognosis for the lung cancer patients. Indeed, a highly significant inverse correlation of higher H19 expression as determined by probe set 217723_x_at with the poorer overall survival of total lung cancer patients was observed (Fig. 5B). To determine whether mdig expression can predict patient survival for other types of cancers, we also analyzed mdig expression and patient survival in breast cancer and ovarian cancer. Interestingly, the prognostic power of mdig was also noted for poorer relapse-free survival of the breast cancer patients (Fig. 5C) and poorer progress-free survival of the ovarian cancer patients (Fig. 5D). These results clearly suggested that the level of mdig expression is an important predictive factor for poorer prognoses of lung cancer, breast cancer and ovarian cancer.

## DISCUSSION

Mdig was first identified as a mineral dust-induced transcript from coal miners' alveolar macrophages by differential display RT-PCR (GenBank BE441202, Jul 25, 2000) [1] and was subsequently found to be a nucleolar protein [4] that might be induced by the c-Myc oncogene [3]. The mdig protein contains a conserved JmjC domain, a signature motif for histone demethylases. However, whether mdig possesses histone demethylase activity remains unclear, despite indirect evidence showing possible contributions of mdig to the demethylation of H3K9me3 in the overexpression or siRNA-based silencing experiments *in vitro*. Because mdig was first identified from patients with occupational lung diseases, whether there is an association between mdig expression and the pathogenesis of human lung cancers remains a compelling question. Our previous reports revealed an elevated level of mdig protein or mRNA in human lung cancers that showed a substantial reduction in the level of H3K9me3 [2], which provided compensatory support to the observations showing decreased levels of H3K9me3 in mdig-overexpressing cells.

Recent studies by Ge *et al*. [16] concluded that recombinant mdig is a protein hydroxylase that catalyzes the histidyl hydroxylation of the ribosomal protein Rpl27a without detectable demethylase activity toward histone proteins. A similar observation was made by screening the JmjC family proteins for possible histone demethylases [14]. There are several possible explanations for the failure to detect the demethylase activity of the mdig protein. First, the recombinant mdig protein generated from the engineered bacteria might lack the modifications that are present in mammalian cells. Such modifications, e.g., glycosylation, may be necessary for determining the histone demethylase activity but not the hydroxylase activity. Second, the demethylase activity of mdig may require assistance from other co-factors in addition to the essential components in a test tube demethylation assay. Third, mdig itself may be not a bona fide histone demethylase but may be able to regulate other histone demethylases in terms of their expression or chromatin binding to influence the methylation status of the histone or other proteins.

To circumvent the pitfalls originating from bacterially-expressed recombinant mdig protein, we applied immunoprecipitation-purified mdig protein from the mdig-overexpressing cells to our demethylation assay using histone H3 peptide. The demethylation reaction was evident due to appearance of the demethylated products following the incubation of mdig protein with the peptide containing H3K9me3 (Figs. 4A and 4B). H3K9me3 is a hallmark of heterochromatin that mostly contributes to the transcriptional silencing of genes by recruiting heterochromatin protein 1 (HP1) for heterochromatin propagation and condensation. The decreased staining signal of DAPI that targets heterochromatin in mdig-expressing cells can be interpreted as an additional sign of mdig's role in H3K9me3 demethylation. However, it should be noted that the demethylation capability of mdig is relatively weak toward bulk histone H3, as evidenced in the immunoblotting experiments. Such a weak activity may reflect two general facts: first, mdig is mostly localized in the nucleoli, which contain a very small portion of the H3K9me3-enriched chromatin; second, mdig is one of many demethylases that are overpowered by a number of methytransferases in a given cell or tissue. Nevertheless, mdig is still capable of reducing the level of H3K9me3 on some specific genes, such as the gene loci of rRNAs, as we reported previously, and H19, as reported here.H19 and the majority of rRNA genes are silenced in differentiated cells by the heterochromatin conformation resulting from DNA methylation and H3K9me3. The demethylation of H3K9me3 will relax the heterochromatin and de-repress the expression of H19 and rRNA that are important for cell growth. Mdig appears to be able to up-regulate the expression of IGF2, macrosatellite X56, c-Myc, and Jhdm3a, which might be achieved through either weakening the heterochromatin structure (IGF2 and macrosatellite X56) or an unexplored, demethylation independent mechanism (c-Myc and Jhdm3a).

H3K9me3 has a fundamental role in gene silencing, DNA repair, the pluripotency of the stem cells, and cell lineage differentiation through heterochromatin compaction. The misregulation of H3K9me3 and heterochromatin conformation have been observed in a number of human cancers [38]. In the mammalian genome, the density of H3K9me3 is high in satellite DNAs, rDNA, imprinted loci, and telomeres that contain multiple repetitive DNA sequences. It is widely accepted that reduced levels of H3K9me3 are associated with genomic instability [39]. Thus, aberrant expression of mdig may influence the integrity of the genome, leading the cells to be prone to malignant transformation. Furthermore, since there are observations indicating a H3K9me3 dependency in the repression of the key stemness genes, most notably the Oct3/4, Nanog and Sox2 that are silenced in differentiated cells [40, 41], the activation or overexpression of mdig may provide a favorable scenario for reprogramming the somatic cells into stem cells or cancer stem cells.

H19 is a developmentally regulated gene that transcribes a lincRNA. The status of allele-specific imprinting and the expression of H19 are determined largely by H3K9me3 and DNA methylation in the ICR and the promoter region of this gene locus. Both H19 lincRNA itself and its derived miR-675 has been linked to oncogenesis [23]. There is evidence showing that H19 can affect the expression profiles of genes involved in epithelial-to-mesenchymal transition (EMT), angiogenesis, and cancer cell metastasis [23]. Furthermore, H19, as a precursor of miR-675, may antagonize the tumor suppressor function of retinoblastoma (RB) through miR-675-mediated translational repression, which was reported to be an important mechanism involved in the pathogenesis of human colorectal cancer [42]. Accordingly, the demethylation of H3K9me3 by mdig and the subsequent de-condensation of the heterochromatin in the ICR and promoter region of the H19 gene will lead to a looping chromatin conformation favoring H19 transcription, which provides the cells with the advantage of proliferation and malignant transformation.

Taken together, our data highlight that mdig is capable of weakening the heterochromatin conformation by reducing the level of H3K9me3, leading to the de-repression of the genes in the heterochromatin region. Our findings also indicate that mdig is indeed a protein with H3K9me3 demethylase activity, which underscores the novel mechanism of mdig in cancer development. It is important to further unravel how the expression and activity of mdig are regulated during the cellular response to extracellular stimuli, such as mineral dust, tobacco smoke, UV radiation, and other environmental carcinogens. In future studies, it would be interesting to explore the feasibility of targeting mdig and other H3K9me3 demethylases as a novel strategy in cancer therapy.

## MATERIALS AND METHODS

### Cell culture

The human lung adenocarcinoma epithelial cell line A549 was purchased from the American Type Culture Collection (ATCC, Manassas, VA). The cells were cultured in RPMI-1640 medium (Invitrogen, Grand Island, NY) supplemented with 5% fetal bovine serum and 1% penicillin-streptomycin (Sigma, St. Louis, MO) in a 37°C humidified incubator in the presence of 5% CO_2_. The cells were dislodged with 0.05% trypsin (Sigma) when they reached 70-80% confluence.

### Cell transfection

For the cell transfection experiments with mdig overexpression and shRNA/siRNA silencing, 5 × 10^5^ cells were seeded into each well of 6-well plates. Cell transfection was performed when the cells reached to 60-70% confluence using Lipofectamine 2000 (Invitrogen). The mdig-GFP expression vector, control vector, RFP-conjugated mdig shRNA, and RFP-conjugated control shRNA were purchased from OriGene (Rockville, MD). Stably transfected clones were established by the addition of puromycin (2 μg/ml) for 2 to 3 weeks. The GFP- or RFP-positive clones were selected under fluorescent microscopy. The overexpression or silencing of mdig was determined by Western blotting. In some experiments, siRNA was used for the transient silencing of mdig. Both the control siRNA and the mdig-specific siRNA were purchased from Qiagen (Valencia, CA). The cells (5 × 10^5^/well) in 6-well plates were reverse transfected immediately during the period of cell seeding by 50 nM siRNA per well and forward transfected 24 h later by 50 nM siRNA per well using the Lipofectamine RNAiMAX reagent (Invitrogen) according to the manufacturer's instructions. After incubating for another 24 h or 48 h, cell lysates were prepared for RNA or protein extraction.

### Western blotting

Cells were lysed using RIPA buffer (Millipore, Billerica, MA) supplemented with phosphatase/protease inhibitor cocktail and 1 mM PMSF through sonication and centrifugation. The proteins in the supernatants were collected and quantified using a Micro BCA Protein Assay Reagent Kit (Thermo Scientific, Pittsburgh, PA). The proteins were boiled in LDS sample buffer (Invitrogen) containing 1 mM dithiothreitol before loading on 10 or 15% SDS-PAGE gels. Following electrophoretic separation, the proteins were transferred onto PVDF membranes (Invitrogen) that were then blocked in 5% non-fat milk/Tris-buffered saline with 0.05% Tween-20 (TBS-T) at room temperature for 1 h. The membranes were incubated sequentially with primary and the second antibodies, followed by image development using CDP-Star Reagent (New England Biolabs, New England, MA) and X-ray film. The primary antibodies applied in the experiments include the antibodies against mdig/mina53, H3K9me3, H3K9me2, H3K9me1, H3K27me3, H3K27me2, H3K27me1, H3K36me3, H3K36me2, H3K36me1, H3K4me3, H3K4me2, and H3K4me1. All antibodies were purchased from Abcam (Cambridge, MA).

### Chromatin immunoprecipitation (ChIP)

ChIP was performed using a Chromatin Immunoprecipitation Assay Kit (Millipore), following the manufacturer's instruction. Briefly, genomic DNA-protein complexes were immunoprecipitated using anti-H3K9me3 antibody or normal rabbit IgG (Santa Cruz Biotechnology, Santa Cruz, CA) as a control. After sonication, the precipitated DNA was amplified by SYBR Green-based quantitative real-time PCR (Roche, LightCycler 480) using primers encompassing the promoter and ICR regions of the H19 gene. The ChIP PCR primers are (the numbers in parentheses indicate the sequence regions corresponding to the GenBank ID AF125183):

5'-CCAGCCATGTGCAAAGTATG-3' (9747-9766)
5'-CCATCCTGGAATTCTCCAAA-3' (9939-9920)
5'-GCGGTCTTCAGACAGGAAAG-3' (9468-9487)
5'-CACGTTCCTGGAGAGTAGGG-3' (9673-9654)
5'-TCTTCAGGTCGGGCATTATC-3' (8112-8131)
5'-GCTGTCCTTAGACGGAGTCG-3' (8290-8271)

### Real-time PCR

Total RNAs were extracted from the cells using TRIzol Reagent and subjected to reverse transcription using SYBR Green High-Capacity cDNA Reverse Transcription Kits (Applied Biosystems, Foster City, CA) using a LightCycler 480 instrument. The primers include: H19 5'-AAAGACACCATCGGAACAGC-3' and 5'-AGAGTCGTGGAGGCTTTGAA-3'; IGF2 5'-TCCTCCCTGGACAATCAGAC-3' and 5'-AGAAGCACCAGCATCGACTT-3'; Jhdm3a 5'-CTGTCCATAAAATATCGAAATACCCTA-3' and 5'-TACAGTATATAAATACATAATTTGGGC-3'; c-Myc 5'-TACCCTCTCAACGACAGCAG-3' and 5'-TCTTGACATTCTCCTCGGTG-3'; and macrosatellite X56 5'-CTCAGGTCTCTTGGCTTTGC-3' and 5'-GTGCGGAGGGTAGAGTGGTA-3'.

### Immunofluorescent microscopy

Cells were seeded in a 24-well plate (6 × 10^4^) in 1 ml medium per well and cultured for 24 h. At the end of culturing, the cells were fixed with 4% formaldehyde in PBS for 15 min at room temperature and washed 3 times for 5 min using PBS, followed by the sequential incubation in blocking buffer for 60 min, primary antibody diluted in Antibody Dilution Buffer at 4°C overnight, and the fluorochrome-conjugated second antibody diluted by Antibody Dilution Buffer for 1 h at room temperature in the dark. After rinsing the cells 3 times for 5 min each, 4',6-diamidino-2-phenylindole (DAPI) (0.1 mg/ml) was added on the top of these cells. The immunofluorescent signal was documented under a fluorescence microscope.

### *In vitro* histone demethylation assay

The *in vitro* histone demethylation assay was performed according to a previous report [32]. Briefly, mdig protein was enriched from Mdig over-expression cell lines through immunoprecipitation using an mdig-specific antibody (Abcam, MA). Trimethyl histone H3K9 peptide (Epigentek, NY) was incubated with the precipitated complex in histone demethylation buffer [50 mM Hepes-KOH (pH 7.5), 70 μM Fe (NH_4_)_2_(SO_4_)_2_, 1 mM α-ketoglutarate, 2 mM L-ascorbic acid] at 37°C for 1-3 hr. For mass spectrum (MS) analysis, the reaction mixture (1 μl) was diluted 20-fold with 0.1% TFA and desalted by ZipTip pipette tips (Millipore, MA). The peptide was eluted 3 times with 4 μl of 30% CH_3_CN/0.1% formic acid, and the collected elution was separated with a linear gradient of 5–80% buffer B (80% ACN and 0.1% FA) over 20 min at a flow rate of 250 nL/min on a C18-reversed phase column (75 μm ID, 15 cm length) packed in-house with ReproSil-Pur C18-AQ μm resin (Dr. Maisch GmbH) in buffer A (0.1% FA). A nanoflow Easy–nLC system (Thermo Scientific) was on-line coupled to the Orbitrap Elite instrument (Thermo Scientific). MS data were acquired using a “Top-20-rCID” data-dependent strategy selecting the fragmentation events based on the precursor abundance in the survey scan (300–1650 Th). Full-scan MS spectra were acquired at a target value of 1 e6 and a resolution of 240,000. The charge state rejection function was enabled; charge states were “unassigned”, and single charge states were rejected.
